# Prevalence of Hepatitis B co-infection amongst HIV infected children attending a care and treatment centre in Owerri, South-eastern Nigeria

**DOI:** 10.11604/pamj.2013.14.89.1711

**Published:** 2013-03-07

**Authors:** Emeka Nwolisa, Francis Mbanefo, Joseph Ezeogu, Paul Amadi

**Affiliations:** 1 Paediatric Infectious diseases Unit, Department of Paediatrics, Federal Medical Centre, Owerri Imo State, Nigeria

**Keywords:** Hepatitis B infection, HIV infection, Children, Immunization, Prevalence, Disease progression, HIV Care and Treatment

## Abstract

**Introduction:**

Hepatitis B infection impacts negatively on disease progression in HIV infected children thereby increasing morbidity and mortality. In spite of the foregoing, there is paucity of data on Hepatitis B co-infection in children living with HIV in Owerri, South Eastern Nigeria.This study set out to determine the prevalence of Hepatitis B Co- infection in HIV infected children attending the Paediatric HIV Care and Treatment clinic of the Federal Medical Centre Owerri.

**Methods:**

The study period was between February and July 2010. Testing for Hepatitis B infection was done using the ACON Hepatitis B surface antigen Rapid test strip. (Acon Laboratories Inc. San Diego.CA).

**Results:**

A total of 139 HIV infected children were enrolled during the study period. The overall prevalence of Hepatitis B Co- infection was 5.8% (8/139). The prevalence in males was 8.2% (5/61) while in females it was 3.8% (3/78). The prevalence of Hepatitis B Co- infection amongst patients on antiretroviral therapy was 4.6%. They accounted for 62.5% of Hepatitis B Co- infection in our study. Previous blood transfusion, gender and age of patient did not show statistically significant relationship with Hepatitis B Co-infection.

**Conclusion:**

Though our study shows a low prevalence of Hepatitis B co infection in HIV infected children in our centre, reduction of the rate is still strongly desirable. Reduction can be achieved by strengthing the uptake of Hepatitis B vaccine as part of the routine childhood immunization programme.

## Introduction

Sub-Saharan Africa continues to bear the brunt of the global epidemic of HIV/AIDS, with two thirds of all adults and children with HIV infection living in the sub-continent [1]. HIV infection in children is still of significant public health importance because of its impact on childhood morbidity and mortality and its attendant negative impact on health services delivery [2]. At the end of 2009, HIV infection accounted for 3% of deaths in children younger than 5 years in Nigeria [2]. Most of the HIV infection in children have been linked to mother to child transmission. Infection could occur during pregnancy, at the time of delivery or post partum through breastfeeding [3].

Sub Saharan Africa is also a region with high prevalence of Hepatitis B infection [4]. Several studies in Nigeria have given varied but significantly high Hepatitis B infection prevalence rates [5–7]. While most of these studies were on adults or the general population, a study in the Niger delta area of Nigeria documented a prevalence of 12.4% amongst children [8].

Given that HIV infection and Hepatitis B infection have similar means and routes of transmission, children who are at risk for one would also be at risk for the other. Newborns who are exposed to HBV and HIV from co infected mothers that do not receive post exposure prophylaxis for HBV have been shown to develop chronic HBV infection [9].

With increased access to antibiotics and antifungal agents, hepatitis viruses, especially hepatitis B and C, are emerging as the leading causes of morbidity and mortality among HIV infected children [10]. This would suggest that the HIV infected child with Hepatitis B co-infection is prone to worse outcome as compared to the child who is only HIV infected.

There is paucity of information within South Eastern, Nigeria on the prevalence of Hepatitis B Co-infection in children who are HIV infected. This study set out to determine the prevalence of Hepatitis B Co- infection amongst HIV infected children attending the Paediatric HIV Care and treatment clinic in our centre in Owerri, South Eastern Nigeria.

## Methods

The Federal Medical Centre, Owerri is a tertiary Health facility in Imo State, South Eastern, Nigeria. It is a multi disciplinary facility providing medical services to clients from Imo State and surrounding States of Abia, Anambra and Rivers. It has an HIV care and treatment centre supported by Family Health International (FHI) through the GHAIN project.

This study was a cross sectional descriptive study, carried out in the Paediatric HIV Care and treatment unit of the clinic from February to July 2010. All HIV infected children ≥18months of age attending the clinic during the study period were eligible. A structured questionnaire was used to obtain demographic information of the subjects, history of previous blood transfusion and information on whether the patient was on Antiretroviral therapy(ART) or not.

Testing for Hepatitis B infection was done using the ACON Hepatitis B surface antigen Rapid test strip. (Acon Laboratories Inc. San Diego.CA). This is a rapid chromatographic immunoassay for the qualitative detection of Hepatitis B surface antigens. The test utilizes a combination of monoclonal and polyclonal antibiodies to selectively detect elevated levels of HBsAg in whole blood, plasma or serum.

The tests and results interpretation were carried out according to manufacturer’s instructions while observing universal precautions.

### Data analysis

Data was analyzed using Statistical Package for Social Sciences (SPSS) Version 15. Prevalence of Hepatitis B co- infection was expressed in percentages. Chi square was used to determine the level of association between Hepatitis B co- infection and specific variables.

### Ethical clearance

Ethical clearance for this study was granted by the Ethics committee of the Federal Medical Centre, Owerri. Informed/Verbal consent was also received from the parents/guardian of the patient before enrollment into the study.

## Results

A total of 139 HIV infected patients were enrolled during the study period. They consisted of 78(56.1%) females and 61(43.9%) males. Female to Male ratio was 1:0.78. Their ages ranged from 18 months to 17 years (Table 1). The overall prevalence of Hepatitis B Co- infection was 5.8% (8/139).

**Table 1:** Age distribution of children enrolled in the study

Age	Number	Percentage
**5 years and less**	54	38.8
**6 years – 10 years**	51	36.7
**11 years and above**	34	24.5
**Total**	139	100

Patients aged between 5years and 10 years accounted for over 75% of those enrolled in the study

The prevalence in males was 8.2% (5/61) while in females it was 3.8%( 3/78). 52 (37.4%) of the patients enrolled in the study had previously received blood transfusion. The prevalence amongst previously blood transfused children was 5.8% (3/52) and amongst those who had never been transfused 5.7% (5/87).

The age group 11 years and above accounted for 62.5% of Hepatitis B infection in our study. The prevalence based on age grouping was also highest in this age group. While it was 14.7% (5/34) for this group, it was 3.7% (2/54) in the group, 5 years and below (Table 2).

**Table 2:** Pattern of Hepatitis B Co-infection based on age

Age	Number Hepatitis B co- infected	Percentage (%)
**5 years and less**	2	25
**6 years – 10 years**	1	12.5
**11 years and above**	5	62.5
**Total**	8	100

Patients aged 11 years and above accounted for over half of the Hepatitis B Co- infection.

The association between Hepatitis B infection and previous blood transfusion (p = 0.940), age (p = 0.062) and Gender of the child (p = 0.274) were not statistically significant (P > 0.05). 108 (Seventy- seven percent) of the children in our study were on antiretroviral therapy, 5 (4.6%) of them were co- infected with Hepatitis B. The prevalence of Hepatitis B Co- infection amongst patients on antiretroviral therapy was 4.6% however, they accounted for 62.5% of Hepatitis B co- infected children in our study.

## Discussion

The prevalence of Hepatitis B Co- infection in our study was 5.8%. This compares with 1.2% reported in Dares salaam [11], 4% in Nairobi [12], 4.9% in a Chinese study [13]. Though higher, the difference between our prevalence and the aforementioned ones, is not expected to be statistically significant giving the limitation posed by our smaller sample size of 139 patients. The prevalence in our study is though less than 19% reported in Maiduguri [14], Northern Nigeria in 2007. The lower prevalence reported in our study, when compared with the earlier report from Maiduguri could be attributed to increasing availability of Hepatitis B vaccine which is now amongst the vaccines that are available in the National Programme on Immunization (NPI) in Nigeria. The other vaccines available in the programme are those against Tuberculosis, Polio, Diphtheria, Tetanus, Measles, Pertusis, Yellow fever and Meninigitis. Hepatitis B vaccine as part of the NPI became available in Nigeria between 2002 and 2003. The lower prevalence of Hepatitis B Co-infection in younger children aged 5 years and less in our study when compared with the older ones aged 11 years and above; who were delivered when Hepatitis B vaccine had not been incorporated nor made routinely and widely available as part of the childhood immunisation programme in Nigeria is supportive of this assertion. A Chinese study has also shown a significant lowering of the prevalence of HBV Infection in children when HBV vaccination coverage increased widely [15].

When blood for transfusion is appropriately screened for Hepatitis B and other transmission transmissible infections (TTIs) the risk of infection is markedly reduced therefore it is not surprising in our study that, the association between previous blood transfusion and Hepatitis B infection was not statistically significant. This finding is similar to that reported by Telatela et al [11] from a Paediatric HIV care and treatment centre in Tanzania. The prevalence of Co- infection was higher in males than females in our study but there was no difference statistically.

The prevalence of hepatitis B co infection amongst patients on ART in our study is low but they constitute Seventy-seven percent of patients with Co infection. The often used first line antiretroviral drug combination in our centre consist of Lamivudine, Nevirapine and Zidovudine. Lamivudine is indicated in the management of both HIV and Hepatitis B infections, as it has been shown to be effective in reducing both HIV-1 and HBV viral replication. However there has been documented resistance to lamivudine which is likely to complicate the course of the HBV disease in HIV 1-infected patients [16, 17]. Prolonged lamivudine therapy can also result in drug-resistant HBV mutants and has been associated with hepatitis flares [18, 19]. Several drug combinations have been advocated to facilitate appropriate and effective management of patients with Hepatitis B co- infection. Suggested treatment options for chronic HBV infection include interferon (IFN), and nucleoside analogues. Use of Lamivudine, adefovir dipivoxil, tenofovir disoproxil fumarate (DF) nucleoside analogues with activity against both HBV DNA polymerase and HIV reverse transcriptase have also been suggested [20–22]. Unfortunately except for Lamivudine, these drugs are not readily available and accessible in resource poor centres like ours and even this is dependent on the benevolence of our supporting partners. Our approach would be better geared towards prevention. Since Hepatitis B vaccine has been shown to significantly reduce the prevalence of Hepatitis B infection, it would appear then that our best option would be to prevent Hepatitis B infection in both HIV infected and un-infected children. Hepatitis B vaccine is one of the antigens offered for administration to children in the National Programme on Immunisation in Nigeria. Presently there are still challenges in its availability as a result of frequent stock outs in vaccination centres located in Primary health care centres in rural areas. Other challenges to vaccine uptake in Nigeria include religious beliefs, erratic and uncoordinated government policies. There are also challenges with storage of the vaccine at the recommended and optimal temperatures giving the poor power infrastructure in most communities in the country. In areas with power, it is often erratic. Attempts at addressing this has commenced with provision of solar panels to provide power for refrigerators used in storage of vaccines. More effort would have to be directed at improving uptake of the vaccine for all newborns. Identifying HIV and Hepatitis B co-infected women in pregnancy is also essential because of the high risk of vertical transmission of HIV/HBV. Administration of Lamivudine to pregnant women has been shown to reduce HBV transmission from highly viraemic mothers to their infants [23]. As lamivudine is already available as part of the national ART program, it can easily be integrated into efforts targeted at preventing/reducing vertical transmission of HBV in HIV/HBV infected mothers.

## Conclusion

Though our study shows a low prevalence of Hepatitis B co infection in HIV infected children in our centre, reduction of the rate is still strongly desirable. This Reduction we believe can be achieved by strengthening the uptake of Hepatitis B vaccine as part of the routine childhood immunization programme.
